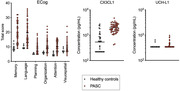# Cognitive functioning and markers of immune and neural dysfunction in Post‐acute sequelae of COVID‐19

**DOI:** 10.1002/alz.093371

**Published:** 2025-01-09

**Authors:** William Cody Reynolds, Isabel Abril, Raymond Quiñones‐Alvarado, Steven E Arnold, Wilfredo F García‐Beltrán, Edmarie Guzman‐Velez

**Affiliations:** ^1^ Massachusetts General Hospital, Boston, MA USA; ^2^ Ragon Institute of MGH, MIT, and Harvard, Cambridge, MA USA; ^3^ Massachusetts General Hospital, Harvard Medical School, Boston, MA USA

## Abstract

**Background:**

Over 65 million COVID‐19 survivors grapple with lasting neurological and cognitive symptoms that persist for months or years after infection, known as neuro‐Post‐acute Sequelae of COVID‐19 (neuro‐PASC). These symptoms are amongst the most common and incapacitating and are hypothesized to represent a heighted risk for neurological disease. Yet, little is known about the pathophysiological mechanisms underlying neuro‐PASC. Here, we examined whether worse neuro‐cognitive functioning was associated with markers of immune and neural dysfunction.

**Methods:**

We report data for 55 people with PASC (average age = 46.09) and 37 healthy controls (HC; average age = 42.84). PASC participants were never in the ICU, 68% female sex, and infected an average of 471.27 days pre‐enrollment. HCs were 57% female sex and 15 were infected an average of 647.73 days pre‐enrollment but fully recovered. We assessed subjective cognitive decline using the Measurement of Everyday Cognition (ECog), memory with the RBANS list learning, and executive functioning with the Trail‐Making Test B (TMT‐B). PASC participants completed a self‐report questionnaire of PASC symptom severity. We measured plasma levels of CX3CL1/Fractalkine, a marker of immune function, and Ubiquitin C‐terminal hydrolase‐L1 (UCH‐L1), a marker of neural function. We conducted t‐tests and linear regressions adjusting for age.

**Results:**

Neurological symptoms were amongst the most severe. Headaches, insomnia, and brain fog were rated as the ones that interfered the most with daily activities. Compared to HCs, PASC participants reported worse subjective memory, language, planning, organization, and attention decline. There were no significant group differences on cognitive test scores. PASC participants exhibited significantly higher CX3CL1/Fractalkine and UCH‐L1, although the latter was not significant when controlling for age. Those with higher levels of CX3CL1 exhibited higher levels of UCH‐L1, indicating a relationship between immune and neural dysfunction. Elevated UCH‐L1 levels were associated with worse TMT‐B performance, but not CX3CL1.

**Conclusions:**

Findings show that neurological symptoms, including subjective cognitive decline, are highly prevalent and severe in PASC. There was clear evidence of heightened inflammation potentially from endothelial cells, and of neural injury, which was associated with worse executive functioning. Therefore, immune dysregulation and neural injury may be underlying neuro‐PASC, and potentially contributing to greater risk for brain disease.